# The dChip survival analysis module for microarray data

**DOI:** 10.1186/1471-2105-12-72

**Published:** 2011-03-09

**Authors:** Samir B Amin, Parantu K Shah, Aimin Yan, Sophia Adamia, Stéphane Minvielle, Hervé Avet-Loiseau, Nikhil C Munshi, Cheng Li

**Affiliations:** 1 Department of Biostatistics and Computational Biology, Dana-Farber Cancer Institute and Harvard School of Public Health, 450 Brookline Ave, Boston, MA, 02215, USA; 2 Department of Medical Oncology, Dana-Farber Cancer Institute, 450 Brookline Ave, Boston, MA, 02215, USA; 3 Hematology Department, University Hospital, Nantes, France; 4 Centre de Recherche en Cancérologie, INSERM U892, Nantes, France; 5 Veterans Administration Boston Healthcare System and Harvard Medical School. 1400 VFW Pkwy, West Roxbury, MA, 02132, USA

## Abstract

**Background:**

Genome-wide expression signatures are emerging as potential marker for overall survival and disease recurrence risk as evidenced by recent commercialization of gene expression based biomarkers in breast cancer. Similar predictions have recently been carried out using genome-wide copy number alterations and microRNAs. Existing software packages for microarray data analysis provide functions to define expression-based survival gene signatures. However, there is no software that can perform survival analysis using SNP array data or draw survival curves interactively for expression-based sample clusters.

**Results:**

We have developed the survival analysis module in the dChip software that performs survival analysis across the genome for gene expression and copy number microarray data. Built on the current dChip software's microarray analysis functions such as chromosome display and clustering, the new survival functions include interactive exploring of Kaplan-Meier (K-M) plots using expression or copy number data, computing survival p-values from the log-rank test and Cox models, and using permutation to identify significant chromosome regions associated with survival.

**Conclusions:**

The dChip survival module provides user-friendly way to perform survival analysis and visualize the results in the context of genes and cytobands. It requires no coding expertise and only minimal learning curve for thousands of existing dChip users. The implementation in Visual C++ also enables fast computation. The software and demonstration data are freely available at http://dchip-surv.chenglilab.org.

## Background

In cancer clinical practice, predicting patient survival based on traditional tumor staging systems using clinical, histopathological and molecular markers remains an integral component in the treatment decision for patients. For example, patients with advanced disease and poor survival prognosis are subjected to more aggressive treatments. However, this conventional approach is non-specific and has limited success in the cancer treatment. Many patients have recurrence despite having aggressive therapy based on survival risk score [1,2].

With high-throughput cancer genomics data, we and others have reported using genome-wide expression signatures to predict survival risk, and these signatures are now increasingly being used in treatment decision for several cancer types [3-6]. Survival predictions have also been carried out using genome-wide copy number alterations [7,8] and microRNAs [9,10]. Encouraged by these results, researchers routinely analyze large sets of microarray data in relation to survival information. Common analysis tasks and endpoints include gene signatures that predict survival risk, survival difference between sample groups defined by unsupervised clustering, and survival analysis using the copy-number data of local genomic regions. Such survival analysis on a high-dimensional data requires statistical programming and command-line skills, or the use of the existing software packages such as BRB-ArrayTools, Survival Online tool and Prediction Analysis for Microarrays (PAM) [11-13].

However, there is no specific utility that can perform survival analysis using SNP array data or draw survival curves interactively for expression-based sample clusters. We have developed the widely-used dChip software that can efficiently process and derive gene expression and copy number data from microarray datasets (http://www.dchip.org) [14,15], and have pioneered using SNP arrays to find chromosomal alterations such as amplification, deletion, and loss of heterozygosity (LOH) [16]. Thus, the addition of survival functions will be helpful for researchers to query and correlate chromosomal regions of interest with associated survival data.

Here, we describe the survival analysis module in the dChip software that performs survival analysis across the genome for gene expression and copy number microarray data. The new survival functions include interactive exploring of Kaplan-Meier (K-M) plots using both expression and copy number data, computing survival p-values from the log-rank test and Cox models, and using permutation to assess the survival significance of copy numbers genome-wide. Researchers can also compare survival curves between sample clustering groups derived from expression data. The dChip survival module enables user-friendly, interactive survival analysis and visualization of microarray data in the context of genes and cytobands. It requires no need for coding and minimal learning curve for existing dChip users. The implementation in Visual C++ also enables fast computation for processing large data sets from studies such as the Cancer Genome Atlas (TCGA).

## Implementation and analysis examples

**Figure 1:**
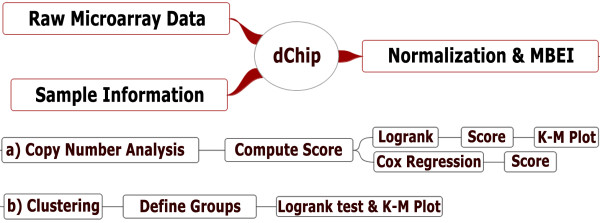
**The overview of dChip survival functions for microarray data**. MBEI: model-based expression index.

The survival analysis functions are implemented in dChip using Visual C++ and optimized for fast computation. The computed log-rank test and Cox model statistics and p-values are confirmed using R code. Figure 1 summarizes the preliminary raw data analysis and new workflow functions in two categories. a) those for SNP copy number data, and b) those for expression-based sample clustering groups.

### Example data sets

Here we will use two example data sets to demonstrate the functions: 1) performing survival analysis using SNP data, and 2) drawing K-M plots using expression-based sample clustering groups. For the first dataset [7], we will discuss the following dChip analysis steps: SNP data input and normalization, plotting copy number data in the chromosome view, carrying out survival analysis using the log-rank and Cox model, and the permutation function to adjust for multiple testing and assess the genome-wide significance of the survival scores. For the second dataset, we will use a gene expression dataset consisting of 170 uniformly treated patients with multiple myeloma with clinical follow-up of more than five years (Munshi et al., manuscript in preparation). We will first perform unsupervised hierarchical clustering and define gene signatures that classify the samples into sub-groups, and then compare K-M curves by the log-rank test among these sub-groups.

### Preparing an example dataset with survival outcome for analysis

We will use a 192-sample microarray dataset processed on Affymetrix SNP 500 K microarray platform [7] to illustrate the usage of the new analysis and visualization functions. This data set represents 192 uniformly treated patients with multiple myeloma (MM). MM is a common type of hematological cancer and characterized by malignant clonal transformation of plasma cells in bone marrow with excess production of a monoclonal immunoglobulin. Chromosomal aberrations are a hallmark of MM with specific changes (del-13 and t(4;14)) giving poor prognosis and other changes (hyperdiploidy and t(11;14)) conferring better survival [17].

We use the dChip software to normalize all the arrays of 192 myeloma samples and additional 10 normal blood samples to compute model-based signal values. Normalization and model-based signals are calculated for each of the two sub-arrays and combined. The median genotype call rates are 96.77% and 97.35% respectively for the 250 K Nsp and Sty sub-arrays. A tab-delimited sample information file is prepared with columns specifying survival outcome including survival time and event indicator (0 = alive, 1 = death). Numerical columns are marked in the column header such as "Survival(numeric)", and will be standardized and displayed above the samples in the clustering or chromosome data views. After loading the normalized data into dChip using the menu function "*Analysis > Open group*", we use the "*Tools > Array List File*" menu function to create an array list to order samples by the values of a particular sample variable. Array list files are also useful when doing survival analysis on a subset of samples, leaving other samples out of the downstream analysis.

**Figure 2:**
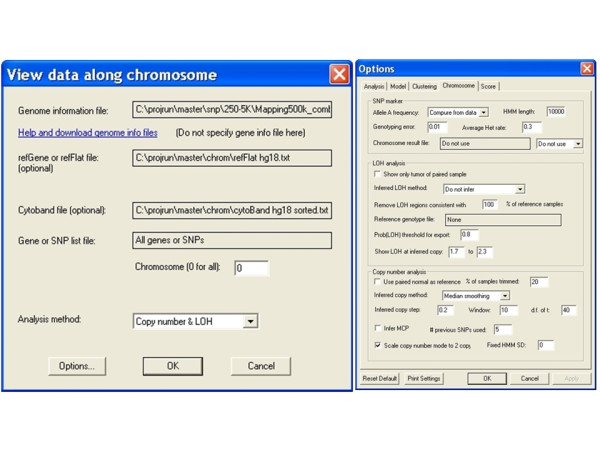
**The *"Analysis > Chromosome" *menu dialog and options to view copy number data along chromosome**. More description of the options is at the dChip website (http://www.dchip.org).

The next step displays the data along chromosomes using the menu function "*Analysis > Chromosome*" to specify analysis parameters for copy number and LOH analysis (Figure 2). The copy number analysis functions are explained in detail in the dChip manual [14]. When the ploidy of samples are unknown (i.e. tumor samples), we can check the option "*Scale copy number mode to 2 copy*" to adjust for ploidy effect sample-wise, so that in hyperdiploid samples the chromosomes with normal copy numbers are estimated to have two copies rather than deletion events.

**Figure 3:**
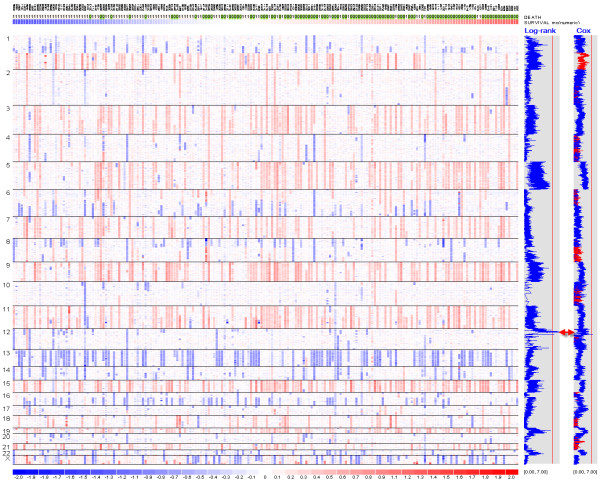
**The dChip chromosome view of copy numbers and survival association scores**. The SNP copy numbers in all the chromosomes are displayed in log2 ratios (red for gains and blue for losses), with SNPs on rows ordered by chromosome and positions, and samples on columns ordered by survival time. Hyperdiploid samples (copy number > 2.5 for a number of chromosomes) tend to locate towards the right, where samples have longer survival. The first blue curve on the right side is SNP-wise, -log10 transformed p-value from the log-rank test, e.g. 3 correspond to a p-value of 0.001. The second blue curve on the right is the Cox model z-scores. The absolute z-scores are displayed SNP-wise: the red color for positive and blue color for negative scores. The vertical red lines indicate the genome-wide score threshold at the significance 0.05 level using permutation analysis (log-rank: 5.71; Cox model: 5.31).

Once the data is displayed by chromosomes, we can toggle between showing individual and all chromosomes using the "*Chromosome > Show All*" menu. Figure 3 shows the gain and loss of copy numbers in the chromosome view, with SNPs on the rows and samples on the columns. It also uses an array list file to sort all the samples by survival time irrespective of the event indicator. We can browse the genome and quickly observe whether copy gain and loss events are associated with survival. Figure 3 shows that hyperdiploid samples with three copies in chromosome 5 tend to locate on the right side (thus longer survival time) rather than on the left side, suggesting hyperdiploidy is associated with better survival outcome of myeloma patients.

### Genome-wide log-rank test using sample groups defined by SNP copy number

**Figure 4:**
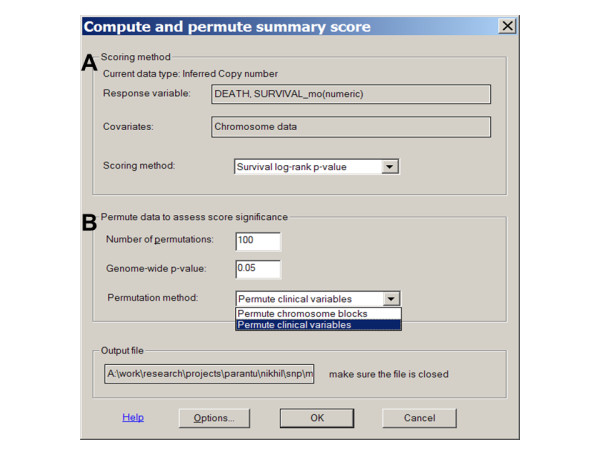
**The *"Chromosome > Compute Score" *menu dialog for log-rank analysis and permutation function**. **(A) **The survival response variables are selected from the data columns in a sample information file. The event and event-free survival (EFS) variables can also be selected to perform survival analysis. **(B) **Additionally, the "Permutation methods" option provides permuting scheme by either "Chromosome blocks" or "Clinical variables".

In the first survival analysis function, we use the menu function "*Chromosome > Compute Score*" to perform genome-wide, single-SNP survival analysis by selecting the "*Survival Log-Rank p-value*" option under "*Scoring method*" (Figure 4). It will perform SNP-wise log-rank test for association between copy numbers and survival outcome. The data variables of either event free survival or overall survival can be selected by clicking "*Response variable*" to open the "*Select factors*" dialog (Figure 4). For a SNP, all the samples are divided into three groups based on this SNP's inferred copy number: Deletion (≤ 1.5 copy), Gain (≥ 2.5 copy), and "No change" (between 1.5 and 2.5 copy). This copy thresholds are adjustable at the "*Options > Score*" dialog. The log-rank test is then applied to the three sample groups, testing the null hypothesis that there is no survival difference between the groups.

**Figure 5:**
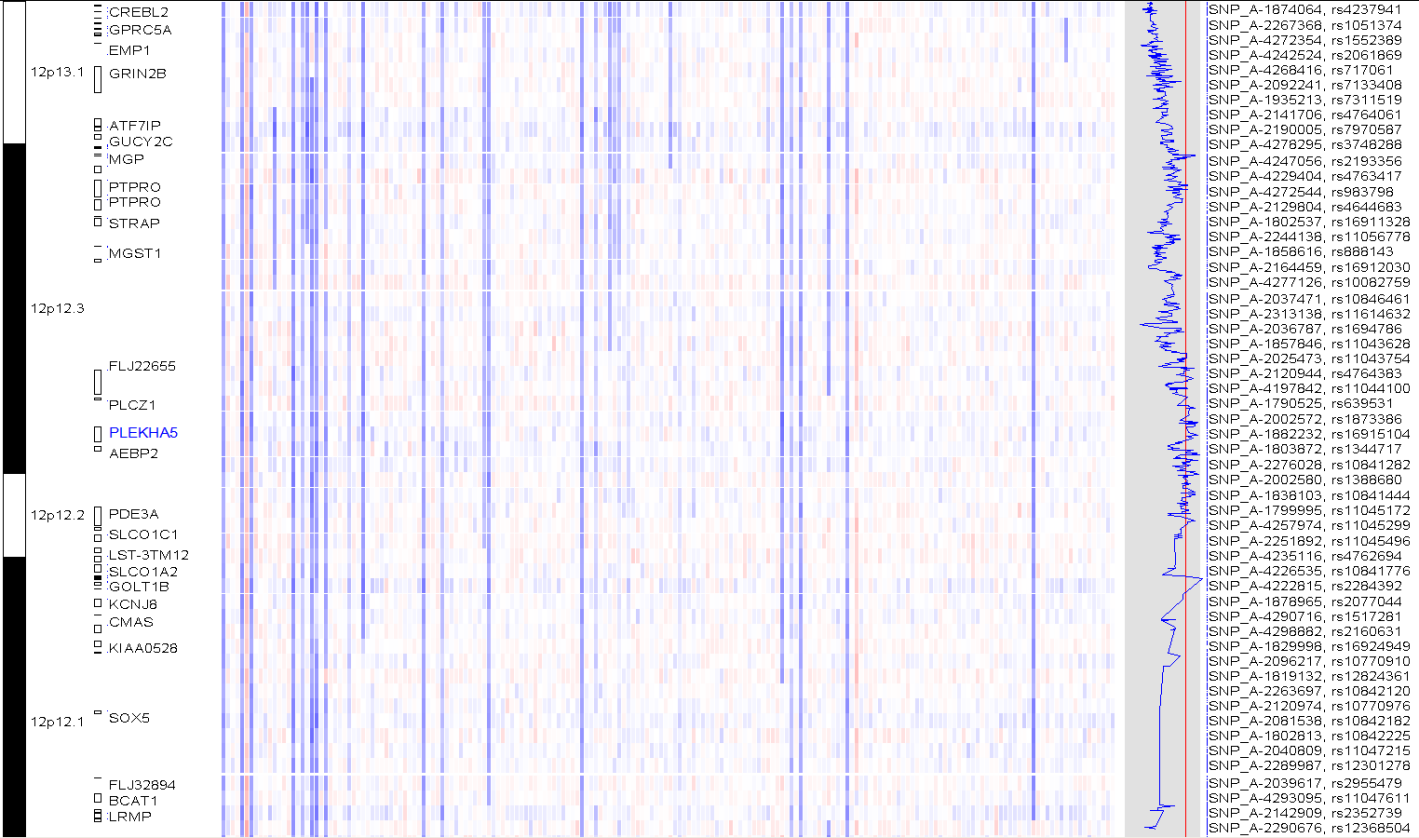
**An enlarged chromosome region**. The enlarged chromosome 12 region has significant survival scores (indicated by the red arrow in Figure 3). The gene names are displayed on the left with bars representing the transcribed region. The Cox z-scores are displayed on the right as the blue curve. One of the SNP in the gene region of PLEKHA5 is used to draw K-M plot (Figure 6).

Across the genome, we compute the log-rank test score for one of every 10 consecutive SNPs to speed up the computation. This is because the inferred copy numbers are correlated for nearby SNPs when computed from 10-SNP local median smoothing, leading to correlated log-rank test scores for nearby SNPs. The window size of 10 is user-adjustable at the dialog option "*Tools > Options > Score > Use one of every 10 markers*". Once the log-rank test is applied to all the SNPs, dChip will display the survival scores genome-wide (Figure 3). The blue curve on the right displays the negative log10 transformed p-values from the log-rank test, with larger values indicating stronger association between copy numbers and survival outcome. The vertical red line in the gray box indicates the score threshold for the genome-wide significance at the 0.05 level by the MaxT permutation method (see the section below on permutation). One or more regions across the genome could have scores exceeding the threshold. Inspecting the genes within these regions could identify candidate survival-associated genes (Figure 5).

**Figure 6:**
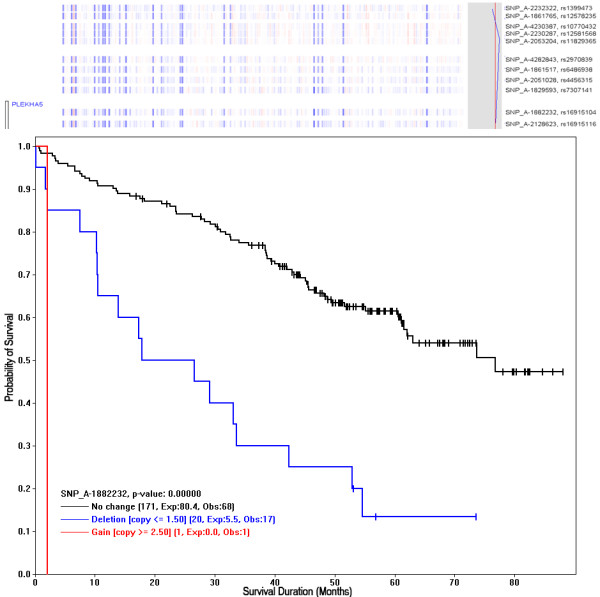
**The Kaplan-Meier plot for a SNP, using its copy numbers to group samples**. The copy number deletion at SNP_A-1882232 (rs16915104 at chromosome 12p12.3) is associated with poor survival. The chromosome view above the K-M plot shows a score value in the right-sided gray box, crossing the significant score threshold (red) derived from permutation analysis. Also, the neighboring SNPs are showing similar survival scores. These SNPs are in the transcribed region of gene PLEKHA5 (PEPP2) - a known oncogenic transcription factor (see text for details). The legend for each sample group shows the number of samples, the Expected events (Exp), and the Observed events (Obs).

To draw a Kaplan-Meier plot (K-M plot) for an individual SNP, we first click on either a SNP nearby a gene of interest (the menu function "*Chromosome > Find Gene*" can search for genes) or a SNP from the regions with significant survival scores. Then, we use the "*View > K-M *plot" function to draw a K-M plot using the copy number data of the SNP to form Deletion, Gain, and "No change" groups as above (Figure 6). The p-value to test the different survival rates between the groups is computed using the log-rank test when there are two or more groups each with 5 or more expected number of events. The SNP (rs16915104) in this K-M plot is within the transcribed region of the PLEKHA5 (PEPP2), an X-linked human homeobox gene at chromosome 12p12.3, which encodes transcription factor with known oncogenic role in cancer and drug resistance via phosphoinositide-mediated signal pathways [18,19]. Using this approach, we can first perform genome-wide screen of SNPs to locate chromosome regions of significant log-rank scores, and then zoom into the peak regions and use K-M plots to check the survival correlation of the SNPs and genes in the region.

### Genome-wide Cox regression using SNP copy numbers

The second analysis function performs univariate Cox regression for survival outcome using the inferred copy numbers of SNPs. After calculating and displaying the inferred copy numbers in the chromosome view, we use the menu function "*Chromosome > Compute Score*" and select the "*Survival Cox Regression*" option under "*Scoring method*" to perform univariate Cox regression for individual SNPs across the genome (Figure 4). The computing time will vary depending on the number of samples and SNPs; it takes less than 10 minutes to process the 500 K SNP, 192 sample data on a 2.4 GHz CPU with 3 GB RAM.

This function displays the absolute z-scores computed from the Cox model in the gray box on the right of the copy number view (Figure 3 and 5). Negative z-scores are displayed in blue, indicating less hazard or longer survival as copy number increases: copy number gains are associated with longer survival, or copy number deletions are associated with shorter survival. Positive z-scores are displayed in red, indicating higher hazard or shorter survivals as copy number increases. As before, ordering samples based on survival time visualizes and confirms the association between copy number alteration events and survival variables. Other continuous or binary sample variables can also be selected as "*Covariates*" to perform multivariate Cox regression to assess the explanatory power of a SNP's copy number to survival in the context of these variables (Figure 4). Permutation can be applied to determine the genome-wide threshold to call chromosome regions significantly associated with survival outcome.

### Permutation to identify significant chromosome regions associated with survival

We also extend the existing permutation function in dChip to assess the genome-wide significance of the survival scores derived from the log-rank test or Cox regression. The number of permutation runs is specified at the "*Chromosome > Compute Score*" dialog (Figure 4). The permutation is computationally intensive but also benefits from computing for only a subset of SNPs across the genome.

The permutation tests the null hypothesis that there is no chromosome region in the cancer genome whose copy number is associated with survival, and therefore any observed association is due to random chance. To simulate data sets under the null hypothesis, we can either permute survival times with censoring indicators across samples or permute chromosome region blocks within every sample. The survival scores from the simulated data sets are then compared to those from the original data set. Specifically for permuting chromosome region blocks, for each sample, whose SNPs are ordered first by chromosomes and then by positions within chromosome, we randomly partition the whole genome into K (≥ 2) blocks, and randomly switch the order of these blocks while preserving the order of SNPs within each block. In this way, the SNPs and their copy numbers in a sample are randomly relocated in blocks to new positions in the genome, while only minimally perturbing the dependence of the copy number data of neighboring SNPs. The same permutation applies to all samples using a different random partition for each sample. The survival score at each SNP locus can then be computed for the permuted data set, and the *MaxT *method can be applied to assess the significance of the original scores [20]. The maximal survival scores from every permuted data set form the score distribution, whose 95^th ^largest value is the genome-wide threshold at the 0.05 significance level to determine the chromosome regions significantly associated with survival in the original dataset. Similarly, clinical variables such as survival times and censoring indicators can be permuted together among all the samples and this achieves similar genome-wide significance threshold (Additional file 1: Figure S1 and Additional file 2: Figure S2).

### Kaplan-Meier plots for expression-based sample clustering groups

Unsupervised hierarchical clustering is frequently used to discover novel sample sub-groups from microarray data or inspect the expression-based samples clusters in relation to clinical variables. In particular, it is interesting to know whether sample clusters correspond to differential survival outcomes.

**Figure 7:**
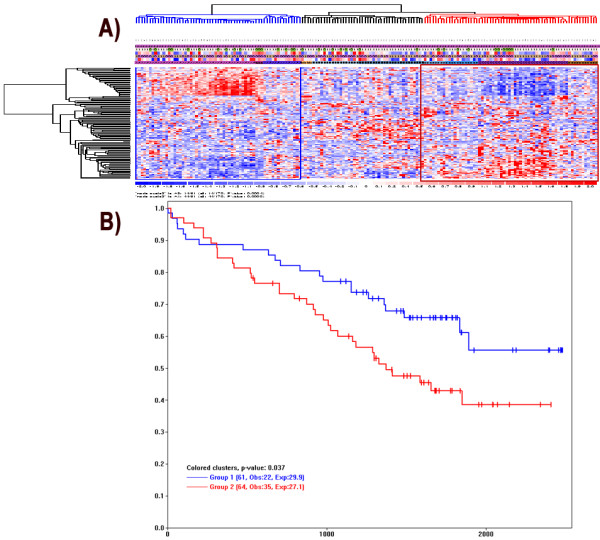
**The Kaplan-Meier survival plots from expression-based sample clusters**. (A) In the sample clustering view from the "*Analysis > Clustering & Enrichment" *function, we can click and select sample clusters of interest for making K-M plots. The blue and red clusters are selected and highlighted. (B) The K-M plot is drawn by the "*View > K-M plot*" function. The legend for each sample group shows the number of samples, the Observed events (Obs) and the Expected events (Exp).

We develop a third analysis function in dChip to facilitate drawing K-M plots based on sample clusters. We first use the "*Analysis > ANOVA & Correlation*" menu to specify the survival variable and event indicator, similar to Figure 4. Next, we use "*Analysis > Clustering & Enrichment*" menu to cluster samples using a variation-filtered gene list. Interested in whether sample clusters correspond to different survival outcomes, we can click to select a main sample cluster branch (in blue color) and use Control-click to select and color additional sample clusters (Figure 7A). We then use the menu "*View/K-M Plot*" to display the Kaplan-Meier plot and log-rank p-value based on the specified sample clusters (Figure 7B). The dChip Analysis View will also show the details of log-rank test and the number of samples omitted due to missing survival data.

## Discussion and conclusions

Gene expression changes and copy-number alterations are known to influence cancer progression and patient survival. Currently, the survival analysis of genome-wide copy number data using SNP arrays and the assessment of significance requires specialized statistical software and skills. We have developed the survival analysis module within the dChip software to streamline the survival analysis and interactive visualization of SNP copy number data and expression-based sample clusters. We use data analysis examples to show how dChip can interactively explore K-M plots and find survival associated genomic regions of interest. The easy user-interface and fast computing make these dChip functions accessible to biomedical researchers.

Survival analysis using copy number data provides options to use either the log-rank test or Cox regression model to compute survival association. The genome-wide view after both analysis methods displays a side-curve showing survival scores for consecutive SNPs. The results from the dChip survival functions agree with previously published results on the same dataset [7]. The amplifications in chromosome 1q and deletions in 1p and 16q are associated with poor survival, whereas the copy number gain of chromosomes 5 and 11 predicts a good prognosis.

We can change the size of SNP window for copy-number smoothing from the default 10 SNPs to other numbers. Increasing the window size will speed up the computation when sample size is large. But we need to be cautious that large window sizes may miss potential survival association of SNPs, especially those SNPs near or within the gene coding regions.

Unsupervised hierarchical clustering is a standard approach to analyzing expression profiling. Researchers are often interested in whether samples clusters correspond to different event-free survival, overall survival, or treatment response outcomes. We have also developed functions in dChip to draw sample cluster-based K-M plots. A user can select two or multiple cluster nodes to compute, display, and export high-quality survival figures. To our knowledge, few software packages provide such graphical interface to ease the survival analysis without coding. We find Survival Online (SO) tool by Corradi et al. [12] a useful online portal for Cox regression and survival analysis using gene expression data. At present, dChipSurv provides similar analysis for SNP array data. Together, these two applications will provide complementary set of features to users in need of survival analysis using expression and copy-number microarray data.

In summary, the dChip survival module addresses the frequent need of many researchers to integrate survival data analysis under a single microarray analysis package with minimal learning curve, fast computing, and no requirement of programming skills. We will add more survival functions as well as extend them to microRNA and RNA-seq data in future dChip versions.

## Availability and requirements

Project name: dChip survival analysis module

Project home page: http://dchip-surv.chenglilab.org/

Operating system(s): Windows 2000 or after

Programming language: Visual C++ 2005

Source code: Available on request.

License: The software is freely available.

## Authors' contributions

CL designed and implemented the software module, S Amin and CL wrote the manuscript, S Amin, PKS and AY contributed to the data analysis and manuscript preparation, S Adamia, SM, HA and NCM designed and performed the microarray experiments.

All authors read and approved the final draft.

## Supplementary Material

Additional file 1 Figure S1 Click here for file

Additional file 2 Figure S2 Click here for file
