# Supplementary material for: The dChip survival analysis module for microarray data
Source: BMC Bioinformatics. 2011 Mar 9;12:72. doi: 10.1186/1471-2105-12-72 (PMC3068974; doi:10.1186/1471-2105-12-72)
Supplement: Additional file 1 — Figure S1 [file 1471-2105-12-72-S1.DOCX]

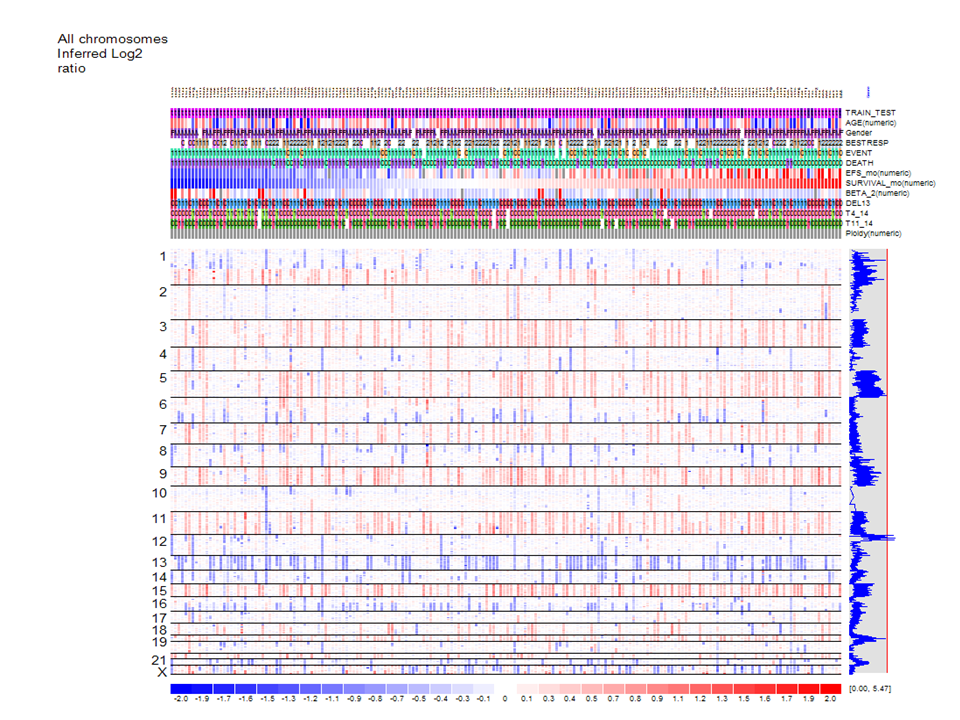


**Additional file 1, Figure S1**

**Title:** Supplementary figure 1a

**Description:**

**Figure caption:** Log-rank survival analysis - Permutation Function: Permuting chromosome blocks. The genome-wide 0.05 significance threshold is 5.47.

**Detailed description:** Figure S1 shows results from the permutation function in dChip to assess the genome-wide significance of the survival scores derived from the log-rank test or Cox regression. The number of permutation runs is specified at the “Chromosome>Compute Score” dialog (Figure 4). The permutation tests the null hypothesis that there is no chromosome region in the cancer genome whose copy number is associated with survival, and therefore any observed association is due to random chance. To simulate data sets under the null hypothesis, here we permute chromosome region blocks within every sample. The survival scores from the simulated data sets are then compared to those from the original data set. Specifically for permuting chromosome region blocks, for each sample, whose SNPs are ordered first by chromosomes and then by positions within chromosome, we randomly partition the whole genome into K (≥ 2) blocks, and randomly switch the order of these blocks while preserving the order of SNPs within each block. In this way, the SNPs and their copy numbers in a sample are randomly relocated in blocks to new positions in the genome, while only minimally perturbing the dependence of the copy number data of neighboring SNPs. The same permutation applies to all samples using a different random partition for each sample. The survival score at each SNP locus can then be computed for the permuted data set, and the MaxT method can be applied to assess the significance of the original scores [20]. The maximal survival scores from every permuted data set form the score distribution, whose 95th largest value is the genome-wide threshold at the 0.05 significance level to determine the chromosome regions significantly associated with survival in the original dataset. The genome-wide 0.05 significance threshold is 5.47 for the example dataset in figure S1.
