# Supplementary material for: The dChip survival analysis module for microarray data
Source: BMC Bioinformatics. 2011 Mar 9;12:72. doi: 10.1186/1471-2105-12-72 (PMC3068974; doi:10.1186/1471-2105-12-72)
Supplement: Additional file 2 — Figure S2 [file 1471-2105-12-72-S2.DOC]

**
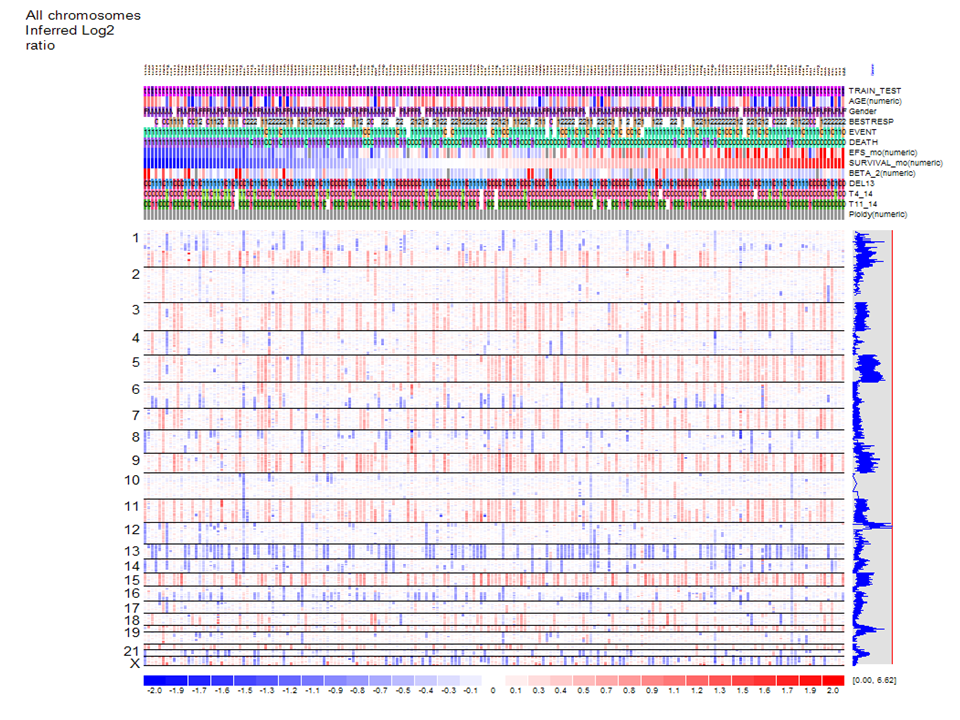
**

**Additional file 2, Figure S2**

**Title:** Supplementary figure 1b

**Description:**

**Figure caption:** Log-rank survival analysis - Permutation Function: Permuting clinical variables. The genome-wide 0.05 significance threshold is 6.62.

**Detailed description:** Similar to figure S2, figure S2 shows results from the permutation function in dChip to assess the genome-wide significance of the survival scores derived from the log-rank test or Cox regression. Here, we permute survival times with censoring indicators across all samples to achieve near similar genome-wide 0.05 significance threshold which is 6.62 for the same example dataset in figure S2.

**Potential reason for larger threshold when permuting with clinical variables instead of permuting with chromosome blocks:**

When permuting sample clinical variables (the survival data), the samples with copy number gains (or losses) at a particular chromosome location can happen to be all assigned longer (or shorter) survival by permutation, leading to high log-rank test scores from the permuted data. In contrast, when permuting chromosome blocks within each sample, the blocks of gains and losses are randomly assigned to other positions in the genome, making high log-rank scores less likely to happen from the permutated data. Nevertheless, the two permutation schemes find similar thresholds that identify chromosome 12p as a region of association to survival.
